# 肺癌的个体化靶向治疗

**DOI:** 10.3779/j.issn.1009-3419.2013.08.10

**Published:** 2013-08-20

**Authors:** Kehua Wu, Larry House, Wanqing Liu, William C.S. Cho, 娟 南

**Affiliations:** 1 Department of Medicine, University of Chicago, Chicago, IL 60637, USA; 2 Department of Medicinal Chemistry and Molecular Pharmacology, College of Pharmacy, Purdue University, West Lafayete, IN 47907, USA; 3 Department of Clinical Oncology, Queen Elizabeth Hospital, Hong Kong, China; 4 天津医科大学总医院，天津市肺癌研究所，天津市肺癌转移与肿瘤微环境重点实验室; 5 香港特别行政区 伊利沙伯医院 临床肿瘤科

**Keywords:** ALK, 生物标记物, EGFR, 肺癌, 新一代测序

## Abstract

由于每一肺癌患者在临床特征、预后、治疗反应和耐受性方面的进展都是独特的，所以肺癌被认为是异质性疾病。个体化用药是指运用标志物来预测哪些患者更易获益于某种治疗。对于肺癌而言，日趋完善的表皮生长因子受体(epidermal growth factor receptor, EGFR)和新发现的棘皮动物微管相关蛋白样4-间变淋巴瘤激酶(echinoderm microtubule associated protein like 4-anaplastic lymphoma kinase, EML4-ALK)是重要的治疗靶标。本综述包括EGFR和EML4-ALK活化的机制、预测性生物标记物、耐药的机理和已有的靶向性酪氨酸激酶抑制剂。本文将通过总结基于生物标记物筛选患者而进行的前瞻性临床试验来探讨EGFR和ALK靶向治疗的疗效。此外，由于革命性的测序和系统生物学技术会为癌症的分子特征提供一个全面的理解，有助于为更适宜靶向治疗的患者提供更精确的区分从而提供更有前景的个体化治疗，本综述也将包括这些技术。同时，非亚裔人群中EGFR和ALK相对较低的突变发生率和突变患者反应的缺乏限制了靶向于EGFR或ALK的治疗的应用。测序和系统生物学策略则可能为这些患者提供新的解决方案。

## 1 引言

在所有癌症中，肺癌死亡率最高，为男性与女性中第二多被诊断出的癌症，分别仅次于前列腺癌和乳腺癌^[1-3]^。已有研究报道，每年有超过160万例新症及130万例死亡^[3]^。最近，美国公布癌症统计数据显示，2011年肺癌新增病例数为221, 130，占所有预计诊断癌症病例的~14%。大约85%-90%的肺癌病例是由于主动或被动(二手)吸烟引起^[4]^。肺癌主要分为两类：非小细胞肺癌(non-small cell lung cancer, NSCLC, ~85%)和小细胞肺癌(small cell lung cancer, SCLC, ~15%)^[1]^。NSCLC可进一步分为鳞状细胞癌(squamous cell carcinoma, SCC)、腺癌和大细胞肺癌(large cell lung carcinoma, LCLC)。遗传易感性在肺癌，尤其是年轻患者的发病中起关键作用^[2]^。

肺癌具有侵袭性，其治疗仍为医疗界最具挑战性的任务之一。常规治疗方法包括手术、放疗和化疗。治疗方法的选择取决于癌症类型(小细胞或非小细胞)、进展阶段和基因特征。诊断为肺癌的患者常接受一种以上治疗方法。小分子抑制剂的发现和发展对NSCLC的治疗产生了重大影响^[5]^。在过去的十年中，有四种分子靶向药物被批准用于治疗肺癌：吉非替尼(2002)、厄洛替尼(2003)、贝伐珠单抗(2006)和克唑替尼(2011)^[6]^。2003年-2006年间肺癌的1年生存率为43%。但是，所有分期的NSCLC的总体5年生存率仍低至16%-17%，SCLC则更低(6%)。尽管早期患者的5年生存率可达53%，但是仅15%的病例在肿瘤尚在早期时被及时发现^[2]^。

由于每一肺癌患者在临床特征、预后、治疗反应和耐受性方面的进展都是独特的，所以肺癌被认为是异质性疾病^[7, 8]^。人类基因组计划的启动催生了癌症治疗的“个体化用药”。这一革命性的方法可帮助患者改善预后并避免不必要的治疗^[8]^。肿瘤学中“个体化用药”一词是指在癌症治疗中，通过鉴别可确切预测哪些患者更易获益于某种治疗的分子标记物，来摒弃“一刀切(one size fits all)”的策略^[9]^。在过去的20年中，靶向治疗研究集中于新型治疗项目。个体化靶向治疗已用于各种癌症的治疗，例如：NSCLC、头颈部鳞癌^[10]^、结直肠癌^[11]^、前列腺癌^[12]^和乳腺癌^[13]^。在将来，肿瘤细胞基因和蛋白的鉴定可协助早期检测，并帮助医生为每一例患者制定最佳靶向治疗方案。

本综述将总结表皮生长因子受体(epidermal growth factor receptor, EGFR)和新发现的棘皮动物微管相关蛋白样4-间变淋巴瘤激酶融合基因(echinoderm microtubule associated protein like 4-anaplastic lymphoma kinase, EML4-ALK)靶标的通路、机制和现有的相关抑制剂。通过汇总基于有效分子标记物的前瞻性临床试验来评估酪氨酸激酶抑制剂(tyrosine kinase inhibitors, TKIs)的疗效。还会探讨革命性的测序和系统生物学策略，通过着眼于全基因组测序和可能的畸变而非单一靶标(如EGFR、KRAS或EML4-ALK)，个体化用药有望成为可能。

## 2 分子靶标

### 2.1 EGFR

在细胞表面受体酪氨酸激酶(receptor tyrosine kinase, RTK)中，EGFR是ErbB家族的一员。EGFR家族包括4个成员：EGFR(或ErbB-1)、HER-2(或ErbB-2)、HER-3(或ErbB-3)和HER-4(或ErbB4)^[14]^。已有研究显示，RTKs通过控制调节增殖和凋亡的信号转导通路在肿瘤发生中起重要作用^[15]^。RTKs(HER-2除外)通过与特定的活化可溶性配体结合被激活，激活发生在RTKs胞外区^[16]^。配体与受体的相互作用可促进功能性活化同源二聚体(EGFR二聚体)或异二聚体(HER3或HER4二聚体)的形成，也可激活细胞内激酶部位及后续受体C-羧基端尾的ATP依赖性交互自身磷酸化^[14, 17]^。最后，磷酸残基募集各种胞质信号分子作为停泊位点，触发包括PI3K/AKT促存活、STAT转录和RAS/RAF/MEK增殖通路在内的下游胞内信号通路(图 1)^[18, 19]^。通过促进凋亡和生长停滞可解除对信号通路的控制^[19, 20]^。在50%-80%的NSCLC患者中EGFR过表达^[21]^，EGFR过表达与血管形成和不良预后相关^[22]^。EGFR变异与发病机理的关系使之成为靶向治疗的主要候选分子。

**1:**
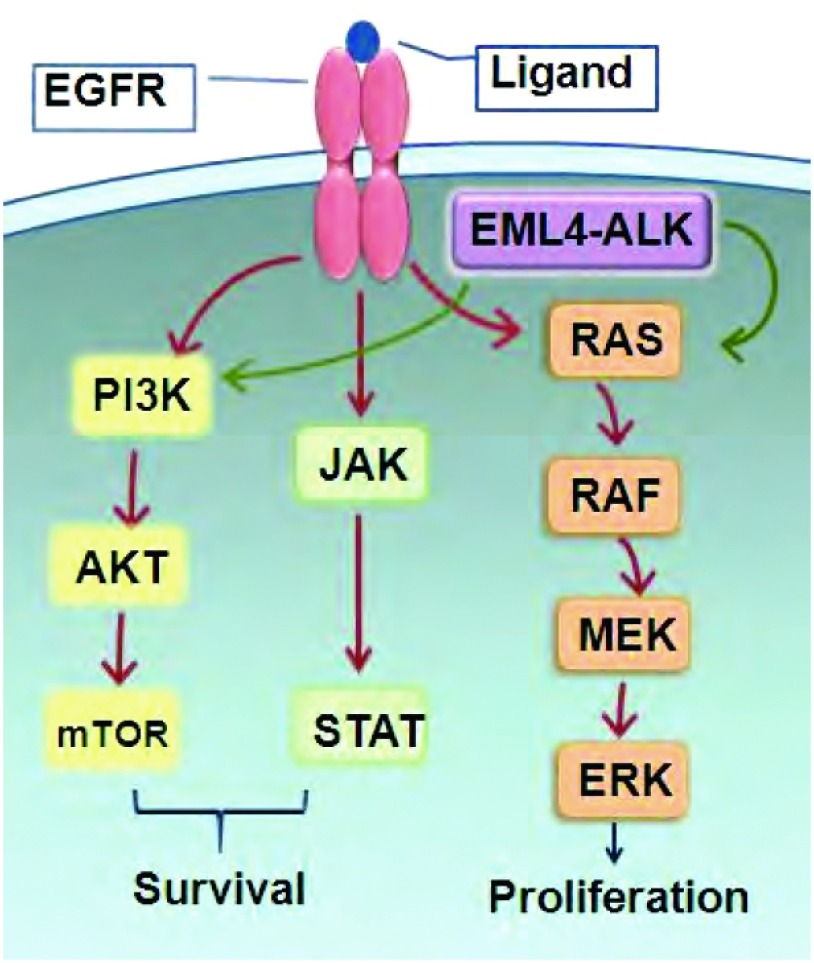
表皮生长因子受体(Epidermal growth factor receptor, EGFR)。EGFR与配体结合可触发包括PI3K/AKT促存活、STAT转录和RAS/RAF/ MEK增殖通路在内的下游胞内信号通路。棘皮动物微管相关蛋白样4-间变淋巴瘤激酶(echinoderm microtubule associated protein like 4-anaplastic lymphoma kinase, EML4-ALK)融合蛋白主要激活RAS/RAF/ MEK和PI3K/AKT通路。EGFR和EML4-ALK信号通路的扩增可促使细胞增殖、细胞活动和致癌作用。

准确的药物反应预测因子可帮助医生预测哪些患者最易获益于EGFR TKIs。这些预测因子或有助于获得最佳治疗且避免耐受。2004年3项有影响力的研究发现EGFR激酶位点的一系列体细胞突变，这些突变与EGFR TKI治疗的反应相关^[23-25]^。至今为止，EGFR突变被认为是EGFR TKI治疗疗效的最强的预测生物标记物^[26]^，因为突变患者的反应率(response rate, RR)更高(37.5%-100% *vs*. 2.9%-23%^[27]^；一线治疗：70% *vs*. 33.2%；二线治疗：47.4% *vs*. 28.5%^[28]^)，总体生存期(overall survival, OS)更长(13-23个月*vs*. 5-17个月^[27]^)。Mok^[29]^总结了6项临床试验来比较携带EGFR阳性突变患者对EGFR TKIs和化疗的反应。患者对EGFR TKIs的反应比化疗好，表现为RR更高(62.1%-84.6% *vs*. 10.5%-47.3%)和无进展生存期(progression-free survival, PFS)更长(8.4-13.1个月*vs*. 4.6-6.7个月)。2011年4月，美国临床肿瘤学会(American Society of Clinical Oncology, ASCO)发布初步临床意见，建议采用EGFR TKI一线治疗新诊断为晚期的NSCLC患者时应基于阳性EGFR突变的检测^[30]^。在非吸烟东亚女性腺癌患者(95%见于腺癌)中EGFR突变尤其常见^[31-36]^。有几项综述汇总了EGFR突变的频率和分布(图 2)^[14, 15, 29, 33, 37-39]^。

**2:**
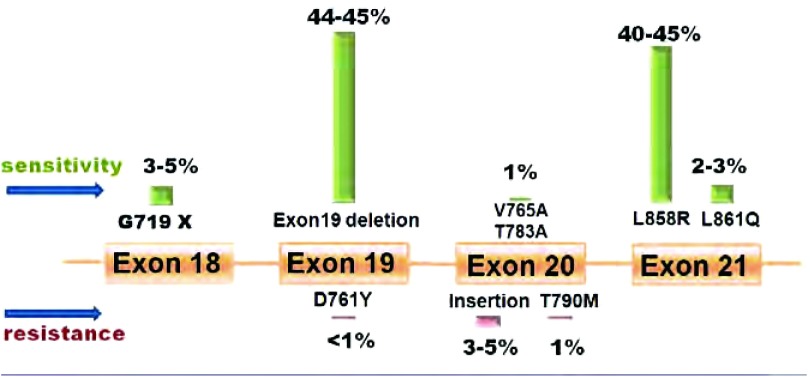
EGFR突变的频率。19外显子缺失位于747-750残基，主要由delGlu746-Ala750、delGlu746-Ser752insVal、delLeu747-Thr751、delLeu747-Ser752和delLeu747-Pro753insSer组成。

EGFR基因拷贝数目也被认为是对EGFR TKI治疗反应的较好预测因子。一些研究显示，EGFR拷贝数目增多与厄洛替尼和吉非替尼的总RR更高、PFS更长和OS获益相关^[40-42]^。事实证明EGFR突变比EGFR基因拷贝数目更具有筛选性^[43]^。

### 2.2 EML4-ALK

ALK酪氨酸激酶受体作为NSCLC新兴的生物标记物和治疗靶标，最近得到更多关注。ALK是位于2号染色体上胰岛素受体家族的一员，编码跨膜受体酪氨酸激酶^[44, 45]^。ALK的激活首先通过融合基因的形成^[47]^。ALK胞内激酶区与EML4的*N*末端融合，然后编码具有激酶活性的胞质嵌合蛋白，随后驱动肿瘤生长^[47]^。NSCLC患者中EML4-ALK重排常见于年轻非吸烟腺癌患者^[47, 50]^。有研究报道，2%-11%肿瘤患者中EML4-ALK呈阳性，SCC中则罕见^[33, 36, 51, 52]^。

### 2.3 KRAS

KRAS突变是EGFR TKIs反应的负性预测因子，主要引起原发耐受^[53]^。肺腺癌中大部分KRAS突变与吸烟有关。KRAS阳性突变只见于NSCLC(主要为腺癌)，与EGFR和ALK突变相互排斥^[54]^。携带KRAS突变的患者不采用EGFR TKI治疗^[55]^。

### 2.4 研发中的潜在靶标

哺乳动物雷帕霉素靶蛋白(mammalian target of rapamycin, mTOR)具有丝氨酸/苏氨酸激酶活性，通过与配体结合可激活PI3K通路，最终调节细胞周期。mTOR抑制剂的研发为实体瘤患者的治疗提供了更多机会。至今，在NSCLC患者中采用mTOR抑制剂单一疗法或联合治疗的研究已进入Ⅰ期/Ⅱ期临床试验。这些mTOR抑制剂包括西罗莫司(sirolimus)、西罗莫司脂化物(temsirolimus)及依维莫司(everolimus)等^[56]^。

纤维细胞生长因子受体1 (fibroblast growth factor receptor 1, FGFR1)的扩增主要见于SCC(高达~20%)，是采用抗FGFR1药物治疗的潜在靶标^[57]^。Dy等报道剂量依赖性肿瘤细胞死亡有赖于采用Y15(1, 2, 4, 5-苯四胺四盐酸盐)进行治疗。Y15是非受体酪氨酸激酶——局部粘着斑激酶(focal adhesion kinase, FAK)的小分子抑制剂^[58]^。DDR2激酶基因突变可驱动SCC。携带DDR2突变的细胞系对阻止细胞转化的达沙替尼敏感。DDR2突变见于4%的SCC^[59]^。

## 3 耐药

已从EGFR TKI治疗获益的部分患者最终会对其产生耐药。TKI治疗的耐药性主要是由于EGFR的T790M位点(位于20外显子)的突变^[60]^。T790M突变占腺癌获得性耐药的50%以上^[33]^。除T790M突变外，MET扩增亦可激活相似的下游通路，占获得性耐药病例的20%^[61]^。

其余30%-40%的EGFR TKIs耐药的潜在机制未明。Ogawa等^[62]^发现，耐药细胞中死亡相关蛋白激酶(deathassociated protein kinase, DAPK)是甲基化的。具有干细胞特性的肿瘤细胞群的产生或许是EGFR TKIs耐药的另一可能原因^[63]^。

与采用EGFR TKI的患者一样，采用ALK抑制剂治疗的患者也会获得耐药。Choi等^[64]^发现，EML4-ALK激酶区域内的二次突变与获得性耐药相伴随。携带C1156Y或L1196M突变的肿瘤细胞对ALK抑制剂的反应较低。与C1156Y突变细胞相比，L1196M突变细胞对克唑替尼更易耐药。PIK3CA基因的突变也是潜在的耐药机制^[65]^。

## 4 靶向药物

阻止EGFR通路的主要方法是与ATP竞争于酪氨酸激酶区域的结合。EGFR TKIs的汇总见表 1。吉非替尼和厄洛替尼是EGFR激酶的可逆性抑制剂，也被称作“第一代”小分子抑制剂。吉非替尼是被食品与药品管理局(Food and Drug Administration, FDA)批准的第一个进入临床试验的靶向药物。吉非替尼“仅可用于已服用该药且医生认为对其有益的癌症患者”^[66]^。新患者不宜服用该药，因为ISEL试验未见OS获益^[67]^。吉非替尼目前在亚洲广泛应用。厄洛替尼作为二线和三线治疗在全球获得批准。采用第一代可逆性EGFR TKIs常在治疗的1年内产生耐药^[68]^，推动了第二代药品的研发(表 1)。第二代TKIs可能通过T790M门控因子突变来克服对厄洛替尼或吉非替尼的耐药。但是，这一功能还需进一步验证，因为有报道称第二代TKI——阿发替尼在防止获得性耐药方面未显现优势^[69]^。一些不可逆性EGFR抑制剂通过中断EGFR成员间的交互信号通路而封闭多种EGFR家族成员，并导致更完全地阻滞。因为达可替尼(PF299804)是EGFR、HER2和HER4的不可逆性抑制剂，所以患者的PFS比厄洛替尼长(*P*=0.017)^[70]^。第二代EGFR TKIs的疗效更好，如延迟耐药，并可用于对可逆性抑制剂耐药的患者。还有多种通路抑制剂处于不同临床阶段，见表 1。

**1:** 用于NSCLC的EGFR TKIs总结

药品	分子特征	批准状态	公司
第一代			
易瑞沙/吉非替尼	EGFR	在超过64个国家销售。它是基于铂类、多西他赛化疗失败后的NSCLC的三线治疗药物。	AstraZeneca
特罗凯/厄洛替尼	EGFR	作为基于铂类化疗失败后的NSCLC的二线和三线治疗药物已被多个机构批准，包括美国食品与药品管理局和欧洲药品管理局。	OSI/Roche/Genentech
埃克替尼	EGFR	中国国家食品药品监督管理局批准用于晚期NSCLC患者的治疗。	浙江贝达药业
第二代			
阿法替尼/BIBW 2992	EGFR/T790M	Ⅲ期临床试验	Boehringer-Ingelheim
达可替尼/PF299804	Pan-EGFR/T790M	Ⅲ期临床试验	Pfizer
来那替尼/HKI-272	EGFR, HER1, HER2	Ⅰ期/Ⅱ期临床试验	Pfizer
AP26113	EGFR/T790M, ALK	Ⅰ期/Ⅱ期临床试验	Ariad
来那替尼/HKI-272	EGFR, HER2	Ⅱ期临床试验	Wyeth
AV412	EGFR, HER2	Ⅰ期临床试验	AVEO Pharmaceuticals
拉帕替尼	EGFR, HER2	Ⅲ期临床试验	GSK
多种信号转导通路抑制剂			
XL647	EGFR, HER2, VEGFR	Ⅱ期临床试验	Exelixis
凡德他尼/caprelsa	EGFR, VEGFR2	Ⅲ期临床试验	AstraZeneca
BMS-690514	Pan-EGFR, VEGFR	Ⅱ期临床试验	Bristol-Myers Squibb
EGFR：表皮生长因子受体；NSCLC：非小细胞肺癌；TKIs：酪氨酸激酶抑制剂。			

2011年8月，克唑替尼用于治疗携带阳性EML4-ALK融合突变的诊断为晚期(局部晚期或转移)的NSCLC得到了FDA的快速批准。相应的诊断方法——Vysis ALK Break Apart FISH Probe Kit也随之获批。AP26113是ALK和EGFR(包括T790M)的有效的双重小分子抑制剂。Ⅰ期剂量递增试验于2011年9月(NCT01449461)启动，按照ARIAD网站信息入选患者的Ⅱ期临床试验有望在2012年年底开始^[71]^。LDK378是选择性的小分子ALK抑制剂。首次人体试验已观察到初步反应^[72]^。其它的ALK抑制剂的总结见表 2。

**2:** 用于NSCLC的ALK抑制剂总结

药品	分子特征	批准状态	公司
Xalkori/克唑替尼	c-MET, ALK	美国食品与药品管理局批准用于携带阳性ALK的晚期 NSCLC患者	Pfizer
AP26113	EGFR/T790M, ALK	Ⅰ期/Ⅱ期临床试验	Ariad
LDK378	ALK	Ⅰ期临床试验	Novartis
AF802/CH5424802	ALK	Ⅰ期/Ⅱ期临床试验	Chugai
ASP3026	ALK	Ⅰ期临床试验	Astrella
X-396	ALK	临床前	Xcovery
GSK-1838705A	ALK	临床前	GSK
NMS-E628	ALK	临床前	-
ALK：间变淋巴瘤激酶；EGFR：表皮生长因子受体；NSCLC：非小细胞肺癌。			

## 5 基于生物标记物入选患者的个体化临床实践

在过去的几十年中，靶向生物标记物的研发、验证、生物标记物的检测、靶向药物的研究和发展以及评估靶向抑制剂疗效的临床前/临床研究有明显进展。借助靶标分层入选患者的前瞻性临床试验最终将使个体化治疗变为现实。

### 5.1 基于患者特性的治疗

最初人们认为患者对EGFR TKIs的反应与临床特征相关，如亚裔腺癌非吸烟女性。有研究关注于评价药物在这些患者中的疗效。Rizvi等^[73]^在富含EGFR突变的患者[吸烟史： < 15包年和/或含有细支气管肺泡癌(bronchioloalveolar carcinoma, BAC)成分]中完成了一项临床试验。符合条件患者的总RR为42%(21/50)，突变患者的RR为81%(17/21)。一项厄洛替尼相关的Ⅱ期试验于近日完成。该研究纳入49例非吸烟或之前很少吸烟的Ⅲb期/Ⅳ期肺腺癌或BAC患者。全部患者的总RR为25.5%，突变患者为66.7%，野生型患者为14.8%。Milella等报道了一项4组(EGFR突变、高多倍体/扩增的EGFR、EGFR和/或pAKT阳性、无吸烟史的腺癌/BAC)患者接受EGFR TKIs作为二线或后续治疗的前瞻性Ⅱ期研究结果。第1组和第4组的总RR最佳和次佳(分别为25%和20%)，第1组和第4组的疾病控制率最高(> 50%)，PFS和OS的差异分别为*P*=0.02和*P*=0.01^[74]^。患者的选择应基于EGFR突变。如无EGFR突变结果，临床特征是第二依据。

### 5.2 EGFR突变患者的靶向治疗

在肺癌中，EGFR是研究最多的靶标。许多临床前和临床研究显示，EGFR突变是接受EGFR TKIs治疗的患者的有效且具有筛选性的预测性生物标记物。包括EGFR突变在内的靶标的发现和验证，使得基于每一例患者独特性的个体化治疗的终极目标越来越近。我们对基于生物标记物入选患者的临床试验进行了总结。

综合生物标记物靶向治疗肺癌(Biomarker-integrated Approaches of Targeted Therapy for Lung Cancer Elimination, BATTLE)的试验是一项验证以生物标记物指导晚期化疗后难治性NSCLC患者治疗的探索性Ⅱ期临床试验^[75]^。BATTLE试验更多地关注于患者，采用实时活组织检查揭示每种肿瘤的独特性，并为医生提供了一种强大的工具来明确哪些靶向治疗更有效。共计255例患者纳入此项研究，首批97例患者随机分配至4组，分别接受厄洛替尼、凡德他尼、厄洛替尼+贝沙罗丁和索拉菲尼。这些符合条件的患者接受11种潜在生物标记物的鉴定。一旦收集了令人满意的基准数的结果，其余158例患者分配至基于其肿瘤类型易于得到更佳反应的治疗组。总体8周疾病控制率(disease control rate, DCR)为46%(厄洛替尼组为34%；凡德他尼组为33%；厄洛替尼+贝沙罗丁组为50%；索拉菲尼组为58%)；中位PFS为1.9个月；中位OS为35%。8周界标分析(landmark analysis)显示，8周疾病控制者的中位生存期为9.6个月，而8周疾病未控制者为7.5个月。BATTLE试验的主要结果显示，基于肿瘤生物标记物给予治疗的患者比未选择治疗的患者更易获益。有效的治疗-标记物-组配对(DCR超过30%的后验概率为0.8)显示，VEGF/VEGFR-2组采用厄洛替尼治疗；EGFR组采用凡德他尼；EGFR、维甲酸X受体、细胞周期素D1、无标记物组采用厄洛替尼+贝沙罗丁；KRAS/BRAF、VEGF/VEGFR-2和无标记物组采用索拉菲尼。针对治疗疗效的单个标记物分析显示，EGFR突变率可以(1)预测对厄洛替尼的反应(*P*=0.04)，(2)预测凡德他尼组VEGFR-2高表达(*P*=0.05)，(3)预测厄洛替尼+贝沙罗丁组细胞周期素D1高表达(*P*=0.01)。这些预测证实了作者的假设，即：生物标记物可以更好地预测8周疾病控制。William Pao评论到“BATTLE试验代表了未来肺癌靶向治疗的基因型驱动研究的重要模式”。综合实时生物标记物检测结果，BATTLE试验促进了为特定患者群制定治疗方案，以达到更满意的个体化治疗。Dr.Edward S Kim说：“最后，我们希望可以先根据肿瘤特征筛选患者，并给予他们恰当的治疗”。后续的BATTLE-2试验正在研究中。

我们总结了近期携带阳性EGFR突变的入选患者的临床试验，见表 3。一些Ⅲ期临床试验比较了EGFR TKIs一线治疗与化疗的疗效和毒副作用(表 3)。比较EGFR突变患者化疗和EGFR TKIs的疗效的前瞻性试验使人们对该人群的恰当治疗有了更深入的了解。这些研究纳入了来自欧洲^[76]^、中国^[77]^和日本^[78]^的携带阳性EGFR突变的NSCLC患者。厄洛替尼或吉非替尼与顺铂/多西他赛或吉西他滨/卡铂进行比较。3项研究中有2项^[76, 78]^发现EGFR TKIs治疗组的PFS明显延长(9.7个月*vs*. 5.2个月，HR 0.37，*P* < 0.0001；9.2个月*vs*. 6.3个月，HR 0.489, *P* < 0.0001)，且RR更高(64% *vs*. 18%)(表 3)。另一项研究^[77]^比较了两组的PFS，厄洛替尼组长于化疗组(13.1个月*vs*. 4.6个月，HR 0.16，*P* < 0.0001)。化疗组比厄洛替尼组引起更多的3级或4级毒性^[76-78]^。3类人群中EGFR TKIs的治疗疗效一致，提示不存在种族差异。EURTAC试验是伴随另外两项研究的第一项前瞻性头对头Ⅲ期研究。它证实了ASCO提议：对NSCLC患者在常规治疗前评估EGFR突变状况^[76]^。LUX-lung 3是在EGFR突变阳性肺癌患者中与培美曲塞/顺铂进行比较的最大型的前瞻性试验，研究显示阿法替尼可能是EGFR阳性患者的有效一线治疗方案，因为PFS得到了改善(11.1个月*vs*. 6.9个月)^[79]^。EGFR突变患者采用EGFR TKIs治疗时，预期反应性比化疗好。由于普遍的交叉或延滞效应，双臂对照研究中OS常无法获得^[29, 80]^。

**3:** 携带EGFR突变的入选患者的临床试验

作者	描述	药物	患者(人数)	终点	结果
在EGFR突变患者中比较EGFR酪氨酸激酶抑制剂(tyrosine kinase inhibitors, TKIs)与化疗的疗效					
Rosell等^[76]^	Ⅲ期(EURTAC)	厄洛替尼*vs*. 顺铂/多西他赛(或吉西他滨)	EGFR^+^(174)	PFS	PFS为9.7个月(厄洛替尼)和5.2个月(化疗) (HR 0.37, *P* < 0.0001)，RR为64%(厄洛替尼)和18%(化疗)。
Zhou等^[77]^	Ⅲ期(OPTIMAL)	厄洛替尼*vs*. 吉西他滨/卡铂(GC)	EGFR^+^(154)	PFS	PFS为13.1个月(厄洛替尼)和4.6个月(GC) (HR 0.16, *P* < 0.0001)。
Mitsudomi等^[78]^	Ⅲ期(WJTOG3405)	吉非替尼*vs*. 顺铂/多西他赛(CD)	EGFR^+^(177)	PFS	PFS为9.2个月(吉非替尼)和6.3个月(CD) (*P* < 0.0001)。
Yang等^[79]^	Ⅲ期(LUX-Lung 3)	阿法替尼*vs*. 培美曲塞/顺铂	EGFR^+^(345)	PFS	阿法替尼组PFS延长(11.1个月*vs*. 6.9个月，HR 0.58，*P*=0.0004)。
Inoue等^[80]^	Ⅲ期(NEJ002)	吉非替尼*vs*. CBDCA+PTX(CP)	EGFR^+^(228)	OS	吉非替尼和CP组间OS无明显差异。中位生存时间和2年生存率分别为27.7个月和57.9% (吉非替尼)、26.6个月和53.7%(CP)(HR 0.887; *P*=0.483)。EGFR TKIs单独给予EGFR突变患者：单臂设计
EGFR TKIs单独给予EGFR突变患者：单臂设计					
Kim等^[81]^	Ⅱ期	一线采用吉非替尼	EGFR^+^(45)	客观RR	客观RR：53.3%；DCR: 86.7%，中位PFS：398天；中位OS：819天。
Tamura等^[82]^	Ⅱ期(WJTOG0403)	一线采用吉非替尼	EGFR^+^(28)	RR	总RR为75%，DCR为96%，中位PFS为11.5个月。
Sequist等^[83]^	II期	一线采用吉非替尼	EGFR^+^(31)	RR	RR为55%，中位PFS为9.2个月。
Inoue等^[84]^	Ⅱ期	一线采用吉非替尼	EGFR^+^(22)	总RR	总RR为66%，DCR为90%，PS改善率为79% (*P* < 0.00005)，68%从PS≥3分的基线改善至PS≤1分。中位PFS、中位生存期和1年生存率为6.5个月、17.8个月和63%。
Sugio等^[85]^	Ⅱ期	厄洛替尼单一疗法	EGFR^+^(19)	-	总RR、DCR、中位PFS和中位生存期为63.2%、89.5%、7.1个月和20个月。
Han等^[86]^	Ⅱ期(ESTERN)	厄洛替尼作为新辅助治疗	EGFR^+^(5)	根治性切除率	一例新辅助化疗后疾病稳定的男性患者接受右上肺叶切除术。
Yang等^[87]^	Ⅱ期(LUX-Lung 2)	一线或二线采用阿法替尼	EGFR^+^(129)	客观RR	客观RR为66%。
Kris等^[88]^	Ⅱ期	一线采用达可替尼(PF-00299804)	EGFR^+^(47)或吸烟 < 10包年、未经tx的腺癌患者。	PFS; PR	EGFR突变患者的PR率为74%。初步PFS为96% (4个月)和77%(1年)。初步PFS为17个月。
基于生物标记物的治疗选择：突变阳性患者采用EGFR TKIs；野生型患者采用化疗					
Zhong等^[89]^	Ⅱ期(LUX-Lung 2)	EGFR^+^臂采用厄洛替尼，EGFR-臂采用GC	EGFR^+^(24)	RR	厄洛替尼臂的RR为58%，GC臂为33%(*P*=0.49)；厄洛替尼臂的RRs为17%，GC臂为25%(*P*=0.64)。
Rosell等^[90]^	Ⅱ期	EGFR^+^者采用厄洛替尼，野生型EGFR者采用化疗联合或不联合顺铂	EGFR^+^(123)		12例EGFR突变患者的中位生存期超过28个月，野生型患者为9个月-11个月。2年生存率分别为73.3%和0%-41.2%。
第二代EGFR TKIs在耐药患者中的疗效					
Pietanza等^[91]^	Ⅱ期	XL647	41例疾病稳定≥12周后进展的复发或再发晚期NSCLC或对厄洛替尼或吉非替尼有反应和/或携带EGFR T790M的患者	客观RR	客观RR为3%，携带T790M的患者中67%疾病进展，未携带T790M的患者中14%疾病进展，11例患者(28%)由于毒性作用需减少剂量。
Sequist等^[92]^	Ⅱ期	来那替尼	167例患者先前采用TKI治疗或EGFR TKIs≥12周	客观RR	EGFR突变患者的客观RR为3%，其他患者为0%。
DCR：疾病控制率；EGFR：表皮生长因子受体；OS：总生存期；PFS：无进展生存期；PS：体能状态；PR：部分缓解；RR：反应率。					

EGFR TKIs一线治疗基于生物标记物入选患者的疗效和安全性得以前瞻性地评估(表 3)。RR范围为53.3%(韩国患者)^[81]^至75%(日本患者)^[82]^。PFS为7.1个月至398天。3级毒副作用的发生率为~13%^[82]^。DCR为86.7%^[81]^至96%^[82]^。其中两项研究观察到OS(819天，17.5个月)^[81, 83]^，约为未经筛选的NSCLC患者采用化疗的2倍^[83]^。中位生存时间为17.8个月和20个月^[84, 85]^。此外，Han等^[86]^发现，对于Ⅲa-N2期NSCLC携带EGFR突变的患者，厄洛替尼作为新辅助治疗是可行的。Inoue等^[84]^研究了体能状态极差的EGFR突变患者对吉非替尼治疗的反应。这是首次报道观察到该人群可获益于吉非替尼。这一有利反应进一步验证了基于生物标记物的治疗选择是切实可行的，且证实了EGFR突变患者可获益于此筛选策略。EGFR突变患者采用厄洛替尼和吉非替尼单一疗法的PFS(表 3)相当(9.7-13.1个月*vs*. 7.1-13.3个月)。RR范围为53.3%至75%，DCR范围为86.7%至96%^[81-88]^。

有两项生物标记物指导的临床研究，在这两项研究中，根据EGFR突变状态，患者被分配至EGFR TKI组或化疗组(表 3)^[89, 90]^。携带EGFR突变的患者接受厄洛替尼或吉非替尼，野生型患者接受包含/不包含顺铂的化疗(依据BRCA1 mRNA水平)。EGFR突变患者比野生型患者对厄洛替尼/吉非替尼的反应好。

Pietanza等^[91]^和Sequist等^[92]^分别报道了对先前治疗已耐受或产生耐受的患者对XL647和来那替尼的疗效，但均未获得阳性结果(表 3)。仍有许多在研的基于生物标记物的临床试验，如PROSE试验^[93]^和UMIN 000005086^[94]^。

### 5.3 野生型EGFR患者的临床试验

有限的EGFR突变率促使研究者实施试验以评估EGFR TKIs对携带野生型EGFR的患者的疗效(表 4)。基于携带野生型EGFR的患者的Ⅱ期临床试验(表 4)，Kobayashi等^[95]^推断：“采用厄洛替尼的EGFR-TKI可能是对细胞毒素化疗耐受的患者，甚至EGFR野生型NSCLC患者的备选方案”。2011年Matsuura等^[96]^实施的临床试验也发现类似结果。厄洛替尼作为三线治疗携带野生型EGFR的患者。尚可接受的RR(15%)和OS(6.7个月)提示厄洛替尼是无EGFR突变患者的潜在三线治疗选择方案。Yoshioka等^[97]^也报道了一项先前接受过1个-3个化疗方案的携带野生型EGFR的日本患者的Ⅱ期临床试验。客观RR低于研究者最初预期。该研究中厄洛替尼对野生型EGFR患者的疗效有限。Garassino等^[98]^比较了厄洛替尼和多西他赛的疗效，发现作为二线治疗，多西他赛优于厄洛替尼。根据上述前瞻性临床研究，EGFR异质性的患者采用EGFR TKIs治疗是有争议的，这与先前的回顾性研究的结论一致。IPASS研究显示，无突变患者采用吉非替尼的疗效较差(HR 2.85, *P* < 0.001)^[100]^。此外，BR.21试验^[101]^发现，与安慰剂组相比，携带野生型突变的患者易获益于厄洛替尼治疗(不显著)。Sharma等^[15]^发现，约10%-20%的无EGFR突变患者采用厄洛替尼后可获得局部缓解。总之，无突变患者采用EGFR TKIs需要更多研究来确认其有效或无效。

**4:** 携带野生型EGFR的特定患者的临床试验

作者	描述	药物和研究设计	患者的筛选	终点	结果
Kobayashi等^[95]^	Ⅱ期	厄洛替尼单一疗法	EGFR-(31)	DCR和PFS	R R、D CR、中位PFS和生存期分别为17.2%、44.8%、2.1个月和7.7个月。
Matsuura等^[96]^	Ⅱ期	厄洛替尼单一疗法	EGFR-(20)	-	总RR为15%，DCR为55%、中位PFS和OS分别为2.1个月和6.7个月。
Yoshioka等^[97]^	Ⅱ期	厄洛替尼单一疗法	EGFR-(30)	客观RR	客观RR为3.3%，18例(60%)患者疾病稳定，中位生存期和中位PFS分别为9.2个月和2.1个月。
Garassino等^[98]^	Ⅲ期(TAILOR)	厄洛替尼*vs*. 多西他赛	EGFR-(211)	PFS	多西他赛组较厄洛替尼组的PFS明显升高(HR 0.70, *P*=0.016)。
Metro等^[99]^	-	厄洛替尼或吉非替尼单一疗法	EGFR-(67)	PFS; OS	中位PFS和OS分别为2.9个月和18.0个月。KRAS突变患者(1.6个月)较野生型患者(3.0个月)的PFS明显缩短(*P*=0.04)。
DCR：疾病控制率；EGFR；表皮生长因子受体；HR：危险比；OS；总生存期；PFS：无进展生存期；PR：部分缓解；RR：反应率。					

Metro等^[99]^在携带野生型EGFR的患者中完成了一项临床试验。该实验观察了KRAS突变状态与对EGFR TKI反应的关系。携带野生型EGFR的KRAS突变组的中位PFS为1.6个月，而KRAS野生型组为3.0个月，说明野生型EGFR和突变型KRAS与抗药性增加相关。此外，KRAS密码子13突变的患者比KRAS密码子12突变的反应更差，提示特定的KRAS致癌基因的替代可能导致不同的敏感性，而且在预测抗药性/敏感性时应予以考虑。

### 5.4 基于KRAS突变筛选患者

Janne等^[102]^在2012 ASCO会议上报告了他们对KRAS阳性患者的临床研究。这是评估KRAS突变的癌症患者采用靶向治疗的临床获益的第一个前瞻性研究。422例接受过化疗的Ⅲb期-Ⅳ期的KRAS突变的NSCLC患者纳入此研究。两个治疗联合方案[司美替尼(AZD6244, ARRY-142866)+多西他赛(SEL/DOC) *vs*. 多西他赛+安慰剂(DOC)]进行了比较。SEL/DOC组的OS更长(9.4个月*vs*. 5.2个月；无显著差异)。SEL/DOC组较单独DOC组患者的RR(DOC 0%，SEL/DOC 37%，*P* < 0.0001)和PFS(DOC 2.1个月，SEL/DOC 2.1个月，P=0.0138)明显更佳，提示KRAS突变患者更易获益于靶向治疗(SEL+DOC)。BATTLE试验则证明KRAS突变肿瘤可获益于索拉菲尼治疗^[75]^。Riely等^[103]^也发现KRAS突变患者采用mTOR抑制剂地磷莫司较安慰剂的PFS更长(4个月*vs*. 2个月，*P*=0.013，HR 0.36)。

### 5.5 基于ALK重排选择患者

ALK抑制剂的发展比EGFR TKIs更快。连续两年ASCO会议中有两个报告涉及PROFILE 1005试验^[104, 105]^。在ALK重排的NSCLC患者中进行的后续全球临床研究显示，克唑替尼可提供高的RR、好的PFS、适度的毒副作用，且患者症状得到改善^[105]^。Shaw等^[106]^进行的Ⅰ期试验也是生物标记物指导的研究。ALK阳性的患者被分配至克唑替尼治疗组和对照组。对照组患者采用一或二线治疗。研究者发现采用克唑替尼治疗的ALK阳性患者较ALK阳性对照组的1-2年OS更高。采用克唑替尼治疗的ALK阳性患者与采用EGFR TKIs治疗EGFR突变组的生存期类似，提示ALK阳性患者与EGFR突变患者一样，可获益于依照其生物标记物选择的方案。

## 6 测序和系统生物学策略

2003年人类基因组计划完成后，全基因组图谱技术取得长足进展，如新一代测序(next-generation sequencing, NGS)，可缩短时间并降低经济成本。技术改进使其可支付得起并很实用，引发“测序和系统生物学策略”^[107]^。这使基于患者基因组信息为合适患者制定恰当靶向治疗的临床决策变得容易。上述特定通路的靶向治疗有赖于一个(或几个)喜好基因。测序和系统生物学策略是通过对肿瘤和正常组织进行全基因组测序、查对癌症的总图谱(global landscape)，随后基于所有的可能畸变靶标选择治疗，对肿瘤采取不同的治疗^[107]^。原发肿瘤和配对转移组织的全基因组测序(whole-genome sequencing, WGS)是对构成肿瘤基础的突变谱的一种新型系统性探索，从而产生了丰富的基因组数据。信息量庞大的基因组数据库为治疗提供了更系统性的考量，因为癌症常与各种基因改变(如结构异常、拷贝数目增加、体细胞单核苷酸变异等)相关。对癌症基因组序列中各种异质性的深入了解有助于建立基于突变的分类、促成个体化治疗^[107]^并预测形成癌症的风险^[108]^。

有几项肺癌相关的WGS研究已完成。一项纳入178例癌组织为SCC的患者的大规模协作研究癌症基因组图谱(Cancer Genome Atlas)鉴定出了30多个明显的体细胞拷贝数目改变的位点。外显子组测序发现13个显著突变基因(假阳性率 < 0.01)。TP53、CDKN2A、PTEN、KEAP1和NFE2L2高表达。mRNA表达谱鉴定出4种不同的表达亚型：NFE2L2和KEAP1突变、FGFR激酶变异、烟草使用率越高总甲基化越多。在20个肿瘤/正常组织配对中，全基因组鸟枪法测序发现几种已知肿瘤抑癌基因的重排，并在RNA测序中得到进一步确认，包括PTEN、RB1、NOTCH1、NF1和CDKN2A。75%的患者(127/178)具有潜在的治疗靶标^[109]^。Lee等^[110]^对一例吸烟15年的51岁男性白种人NSCLC患者的原发性肺癌和相应正常样本进行了NGS。鉴定出不表达基因和启动子区域中的50, 000余个单核苷酸变异，占WGS数据的17.7%。研究者证实了530个变异，其中一个位于KRAS原癌基因，391个变异位于编码区，43个变异为结构改变。Ju等^[111]^报道了对一例年轻非吸烟腺癌患者的配对癌/正常组织的大规模平行全基因组和转录组测序的综合分析。该研究发现，KIF5B和RET的融合可能产生NSCLC的子集，提示嵌合癌基因可能成为诊断与个体化治疗的潜在有前景的分子靶标。WGS方法亦可探索预后指标。Belvedera等^[112]^建立了计算指数(GH指数)，其源自全基因组拷贝数目分析，用以评估总体基因组损伤。GH指数具有预测SCC患者预后的潜在价值，并可用来对患者进行更好地分层。

全基因组图谱为更精心地设计临床试验和更清楚地制定个体化治疗提供了良机。它还存在于几种后勤挑战(logistical challenges)中，包括外显子组和全基因组测序的选择、真正的驱动子和干扰性旁路的区分、对基因组数据进行解释的生物信息学支持和最终的临床执行^[113]^。鉴于时间和成本效益考虑，最近采用该技术的研究多为对多种常见肿瘤的国际性合作研究^[108]^。

## 7 展望

总之，随着新型分子靶标的发现和识别以及明确地抑制癌细胞活性的新型治疗的出现，肺癌分子图谱得到快速发展。从靶标的发现到基于生物标记物的治疗的选择的综合流程使个体化治疗成为现实。大量证据显示携带EGFR突变和ALK重排的患者可获益于相应抑制剂的治疗。本综述中，我们总结了基于入选患者的业已完成的临床实践。这些临床试验显示了有前景的RR、理想的PFS和适度的副作用，说明生物标记物指导的选择策略对阳性突变的患者是可行的。多数临床试验研究了厄洛替尼与吉非替尼。第二代EGFR TKIs和新兴的ALK抑制剂尚未得到充分验证。

另一方面，EGFR和EML4-ALK抑制剂的局限性亦不容忽视。EGFR TKIs治疗携带阳性突变患者的RR为50%-75%。对目标人群缺乏反应的根本原因尚不明瞭，根本原因的了解将有助于极大地提高靶向治疗的疗效。EGFR和EML4-ALK突变分别占美国非吸烟人群的~28%和~11%，在吸烟者中更低。无这些突变的患者(~87%)将不能获益于这些特殊的抑制剂。这一现象限制了癌症治疗的整体进程。而且，活性靶标主要见于NSCLC中的腺癌。针对SCC、LCLC和SCLC的靶向治疗有待研发，以更广地影响NSCLC。全基因组技术和相应的“测序和系统生物学策略”对目前的形势至关重要。该技术有助于我们更全面地了解癌症的分子特征，有助于我们更智能地对患者进行分层并为合适的患者制定靶向治疗，最终实现令人满意和有前景的个体化治疗。

因为仍有许多在研的研究和临床试验，我们期望更多潜在的靶标和标记物被发现和验证，并希望更多的靶向药物在不久的将来被研发。

## Conﬂicts of Interest

Te authors declare no conﬂict of interest.
